# Dynamics and lifetime of persistent activity states in random networks of spiking neurons with strong synapses

**DOI:** 10.1186/1471-2202-14-S1-P121

**Published:** 2013-07-08

**Authors:** Birgit Kriener, Håkon Enger, Tom Tetzlaff, Hans Ekkehard Plesser, Marc-Oliver Gewaltig, Gaute T Einevoll

**Affiliations:** 1 Department of Mathematical Sciences and Technology, Norwegian University of Life Science, Ås, Norway; 2 Inst. of Neuroscience and Medicine (INM-6) and Inst. for Advanced Simulation (IAS-6), Jülich Research Centre and JARA, Jülich, Germany; 3 Blue Brain Project, École Polytechnique Fédérale de Lausanne, Lausanne, Switzerland

## 

The selective persistent activation of populations of neurons in the prefrontal cortex during active memory tasks is one of the best-studied neural correlates of a higher cognitive function, the so-called working memory. Yet, the respective roles of network and neuron properties in this activity are not fully understood. Spiking neuron network models often rely on a selective excitatory connectivity and generate spike trains that are more regular than what is experimentally observed, thus necessitating additional assumptions, such as intrinsic cellular bistability or additional external noisy input.

Recently, it was shown that very simple random networks of excitatory and inhibitory spiking neurons can generate persistent asynchronous-irregular activity only by admitting a certain fraction of comparably strong synaptic weights [1,2]. A large number of experiments showed that synaptic weight distributions are indeed commonly characterized by a large fraction of weak weights and a heavy tail of quite strong weights [e.g. 2,3]. Here, we demonstrate how including such strong synaptic couplings leads to the occurrence of a firing-rate attractor at moderate rates with highly irregular individual spike trains.

Based on the firing-rate transfer of the network, we show that the firing-rate dynamics becomes bistable if the synapses are sufficiently strong: in addition to the quiescent state, a second stable fixed point at moderate firing rates emerges by a saddle-node bifurcation. However, the population firing-rate is characterized by pronounced inherent fluctuations that perpetually perturb this fixed point. It is the trade-off between the size of the population fluctuations and the size of the basin of attraction that thus determines the onset and lifetime of persistent activity states. Moreover, individual neuronal activity turns out to be very irregular, switching between long periods of low firing rate to short burst-like states. We show that this is an effect of the strong coupling strength in the network combined with the finite memory time constant of the neurons. Thus, such irregular neuron dynamics can be a pure network phenomenon, and do not require intracellular bistability or additional high-variability noise as previously suggested.
